# Clinical advances and challenges associated with TCR-T cell therapy for cancer treatment

**DOI:** 10.3389/fimmu.2024.1487782

**Published:** 2024-10-08

**Authors:** Jianing Li, Yongsheng Zhang, Tong Fu, Guoli Xing, Hongbo Cai, Kaiqing Li, Yutong Xu, Ying Tong

**Affiliations:** ^1^ Heilongjiang University of Chinese Medicine, Harbin, China; ^2^ School of Mechanical and Electrical Engineering, Harbin Institute of Technology, Harbin, China; ^3^ Brandeis University, Waltham, MA, United States; ^4^ First Affiliated Hospital, Heilongjiang University of Chinese Medicine, Harbin, China

**Keywords:** TCR-T cell therapy, cancer immunotherapy, clinical trials, genetic engineering, cancer, therapeutic resistance

## Abstract

**Background:**

T cell receptor (TCR)-T cell therapy is an innovative form of cancer immunotherapy that genetically modifies patients’ T cells to target and destroy cancer cells. However, the current status of clinical trials of TCR-T cell therapy for the treatment of cancer remains unclear. This study aimed to comprehensively analyze the registration trials related to TCR-T cell therapy for the treatment of cancer.

**Methods:**

A comprehensive search was conducted in the Trialtrove database for all clinical trials related to TCR-T cell therapy registered by August 1, 2024. Inclusion criteria focused on trials targeting TCR-T cell therapy for oncology, and excluded observational studies and incomplete data. Statistical analysis was performed on key trial characteristics, with between-group comparisons utilizing chi-square or Fisher’s exact tests.

**Results:**

Analysis of 174 eligible clinical trials revealed that TCR-T cell therapy exhibits significant efficacy across various tumor types, particularly in refractory hematologic malignancies and certain solid tumors. Additionally, combining TCR-T cell therapy with other immunotherapies enhanced these anti-tumor effects.

**Conclusion:**

TCR-T cell therapy holds substantial promise for cancer treatment. Future research should focus on optimizing treatment protocols, enhancing efficacy, and minimizing prices to fully realize the potential of this therapy.

## 1 Introduction

Cancer is a heterogeneous group of diseases marked by the loss of normal cellular control, rapid cell proliferation, and the potential to invade other areas of the body, leading to widespread metastasis (1). Globally, cancer remains a major cause of mortality; its incidence continues to rise owing to the increase in the aging population and growing prevalence of unhealthy lifestyles (2). Therefore, effective cancer treatment strategies are essential to reduce mortality and improve survival rates.

Currently, primary cancer treatments include surgery, radiotherapy, and chemotherapy, which remain the most effective and curative options for early-stage cancers such as liver cancer, breast cancer, and acute lymphoblastic leukemia (ALL) (3). Early intervention with traditional therapies can result in favorable outcomes and even cure in some cases (4). However, as cancer progresses and exceeds the therapeutic scope of these conventional treatments, alternative strategies such as immunotherapy are increasingly applied, particularly in the case of advanced-stage patients who are no longer candidates for surgery or when other treatment options have been exhausted (5). For cancers such as brain cancer, where surgical resection may not be feasible due to the location of the tumor, or when the diagnosis occurs at a late stage, immunotherapy offers a potential avenue for treatment (6).

Among them, TCR-T cell therapy, a type of immunotherapy based on genetic engineering modification, is a promising treatment. TCR-T cell therapy involves genetically engineering a patient’s T cells to express specific TCRs that recognize tumor-associated antigens presented by the major histocompatibility complex (MHC) on cancer cells (7). Unlike chimeric antigen receptor (CAR)-T cell therapy, which targets surface antigens independent of MHC, TCR-T cell therapy leverages the natural TCR-MHC interaction to target a broader range of intracellular antigens, including neoantigens derived from mutated proteins, providing a significant advantage in treating solid tumors (8). Furthermore, compared to small-molecule inhibitors, which typically target specific proteins or signaling pathways within cancer cells, TCR-T cell therapy offers the ability to target tumor cells more selectively by recognizing specific peptide-MHC complexes that are often unique to cancer cells. Small-molecule inhibitors, while effective in inhibiting key oncogenic pathways, are often associated with the development of resistance due to mutations in the target protein or compensatory activation of alternative pathways. In contrast, TCR-T cell therapy can better manage tumor heterogeneity by recognizing a broader range of tumor-specific peptides presented by cancer cells, thereby reducing the likelihood of resistance development (9). Compared to traditional cancer therapies, TCR-T cell therapy offers several notable advantages. First, TCR-T cell therapy is highly specific, as it is designed to target patient-specific tumor antigens, which minimizes collateral damage to normal tissues (10). Second, TCR-T cell therapy provides a durable immune response, with the modified T cells able to survive and function in the body for extended periods (11). Third, TCR-T cell therapy has the potential to overcome resistance in certain tumors that are unresponsive to conventional treatments and has shown significant efficacy in treating both hematological malignancies and certain solid tumors (12). This promising therapy is now moving from the research stage into practical clinical applications.

In recent years, TCR-T cell therapy has made significant progress in clinical treatments. Afamitresgene autoleucel (trade name Tecelra), developed by Adaptimmune, is the first FDA-approved TCR-T cell therapy targeting MAGE-A4 for the treatment of advanced synovial sarcoma, marking a major milestone for TCR-T cell therapy (13). Additionally, several TCR-T therapies targeting the cancer-testis antigen NY-ESO-1 have shown significant potential, such as TAEST-16001, developed by Guangzhou Xiangxue Pharmaceuticals, and letectresgene autoleucel, developed by Adaptimmune. These have demonstrated positive results in clinical trials for advanced soft tissue sarcoma and liposarcoma, respectively (14, 15). Other ongoing studies include a TCR-T therapy targeting EBV, developed by TCRCure Biopharma, for metastatic head and neck cancer and cervical cancer, as well as TC-510, developed by TCR2 Therapeutics, which targets mesothelin for the treatment of late-stage mesothelin-expressing cancers (16). These studies suggest that TCR-T cell therapy has the potential to become the next breakthrough in cancer treatment.

However, TCR-T cell therapy still faces some limitations. First, TCR-T cell therapy usually involves complex biotechnology and individualized treatment protocols, making the treatment very expensive and a huge financial burden for many patients (17). Second, tumor cells may exhibit different genetic and phenotypic characteristics in the same patient. This heterogeneity makes it difficult for a single therapy to be effective against all tumor cells (18). Therefore, an in-depth analysis of the mechanism of action of TCR-T cell therapy in different cancer types, the results of clinical trials, and its limitations is of great clinical significance. Ongoing research and clinical trials continue to expand our understanding of TCR-T cell therapy, striving to overcome these challenges and unlock its full potential in cancer treatment. We utilized the Trialtrove database, which aggregates global clinical trial data and offers a robust platform for evaluating the latest advancements in TCR-T cell therapy (19). to obtain the most current and comprehensive clinical trial information regarding these trials, enabling us to evaluate the efficacy, safety, and clinical progress of TCR-T cell therapy across different trial phases. This in-depth analysis not only offers a thorough understanding of the therapeutic potential of TCR-T cell therapy but also supports future research by providing evidence-based insights into its clinical applications and outcomes.

## 2 Methods

### 2.1 Data source and selection criteria

This study used data from the Trialtrove database, which compiles information on clinical trials globally. We searched for all trials registered up to August 1, 2024, related to TCR-T cell therapy for cancer treatment. The search term used was “Therapeutic class: ‘Cellular therapy, T cell receptor’ Therapeutic area: ‘Oncology’.” To guarantee the relevance and reliability of the data, only interventional studies were selected for inclusion.

### 2.2 Inclusion and exclusion criteria

The selection process for this study was guided by clearly defined inclusion and exclusion criteria. Inclusion criteria involved trials explicitly focused on TCR-T cell therapy, with clearly stated therapeutic targets or mechanisms of action related to cancer treatment. Exclusion criteria were more nuanced, targeting trials that lacked critical information (such as undefined drug targets or mechanisms), contained incomplete datasets, or involved non-interventional study designs (e.g., observational studies or registries that did not directly assess therapeutic outcomes). Trials with missing or ambiguous drug targets were subjected to additional review. If the trial lacked sufficient detail to determine the therapeutic target, it was excluded from the final analysis.

### 2.3 Handling incomplete data

Incomplete data were handled systematically. Trials missing substantial portions of information, such as primary or secondary outcomes, were excluded to ensure the robustness of our analysis. In cases where trials had unknown or ambiguous drug targets, these were excluded unless further cross-referencing with external databases or published sources clarified the therapeutic approach. This method minimized bias and maintained the integrity of the dataset by ensuring that only well-defined, target-specific trials were included in the analysis.

### 2.4 Data extraction and statistical analysis

Data extraction was meticulously conducted by two independent investigators, each of whom followed a predefined protocol to ensure consistency and accuracy. The extracted data were then cross-verified against multiple published databases to ensure the integrity of the information. The key characteristics of the included trials were documented in a structured table, ensuring a comprehensive overview of the study parameters. Descriptive statistical methods were used to summarize the trial characteristics, with categorical data reported as frequencies and percentages. Group comparisons were conducted using the Pearson χ² test or Fisher’s exact test when the number of trials in a category was fewer than 10. A two-sided p-value of < 0.05 was considered statistically significant. All analyses were performed using SPSS software.

## 3 Results

### 3.1 Trial characteristics and funding sources

**Figure 1:**
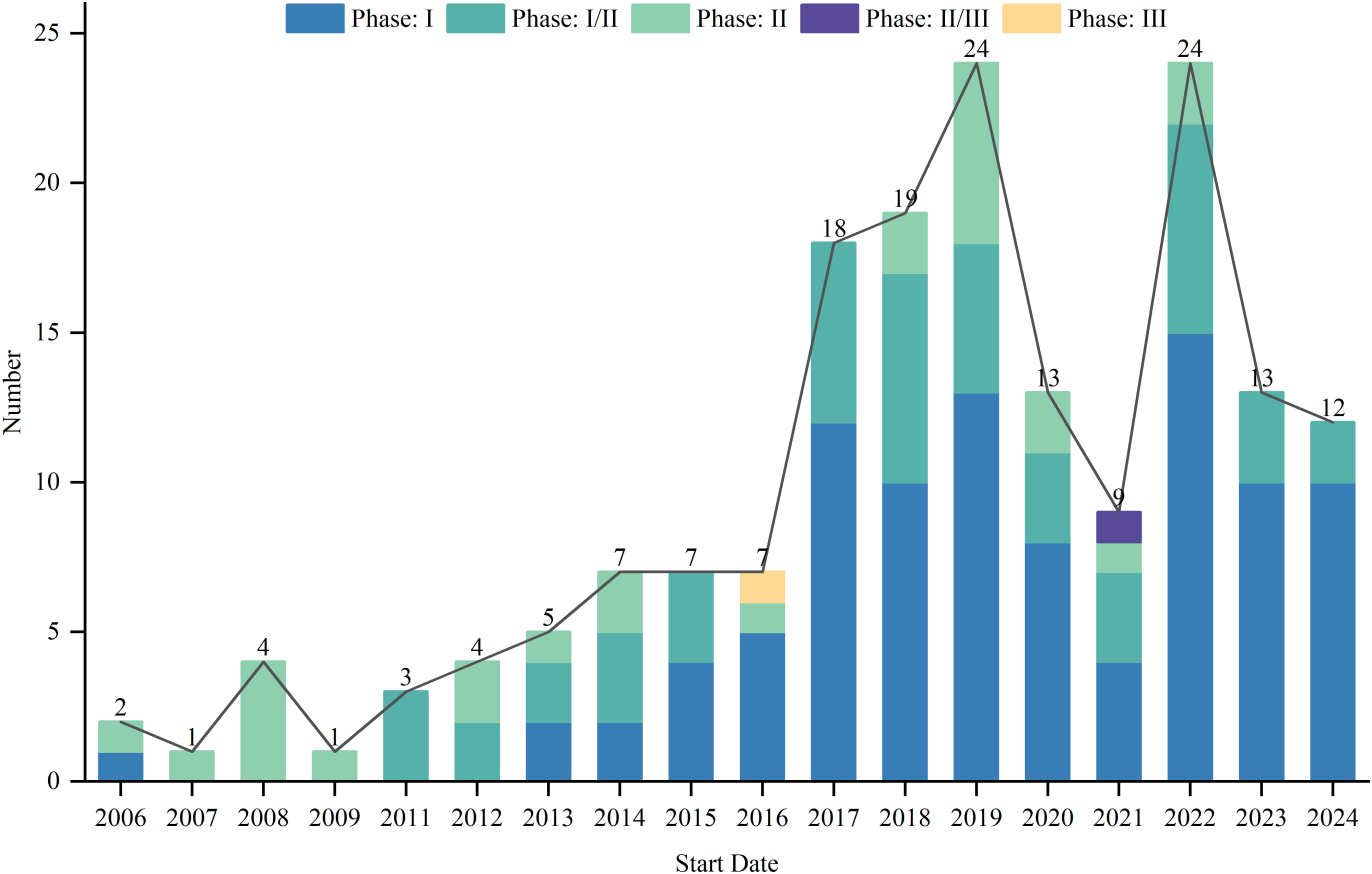
Start date distribution of clinical trials for TCR-T cell therapy in cancer treatment.

**Figure 2:**
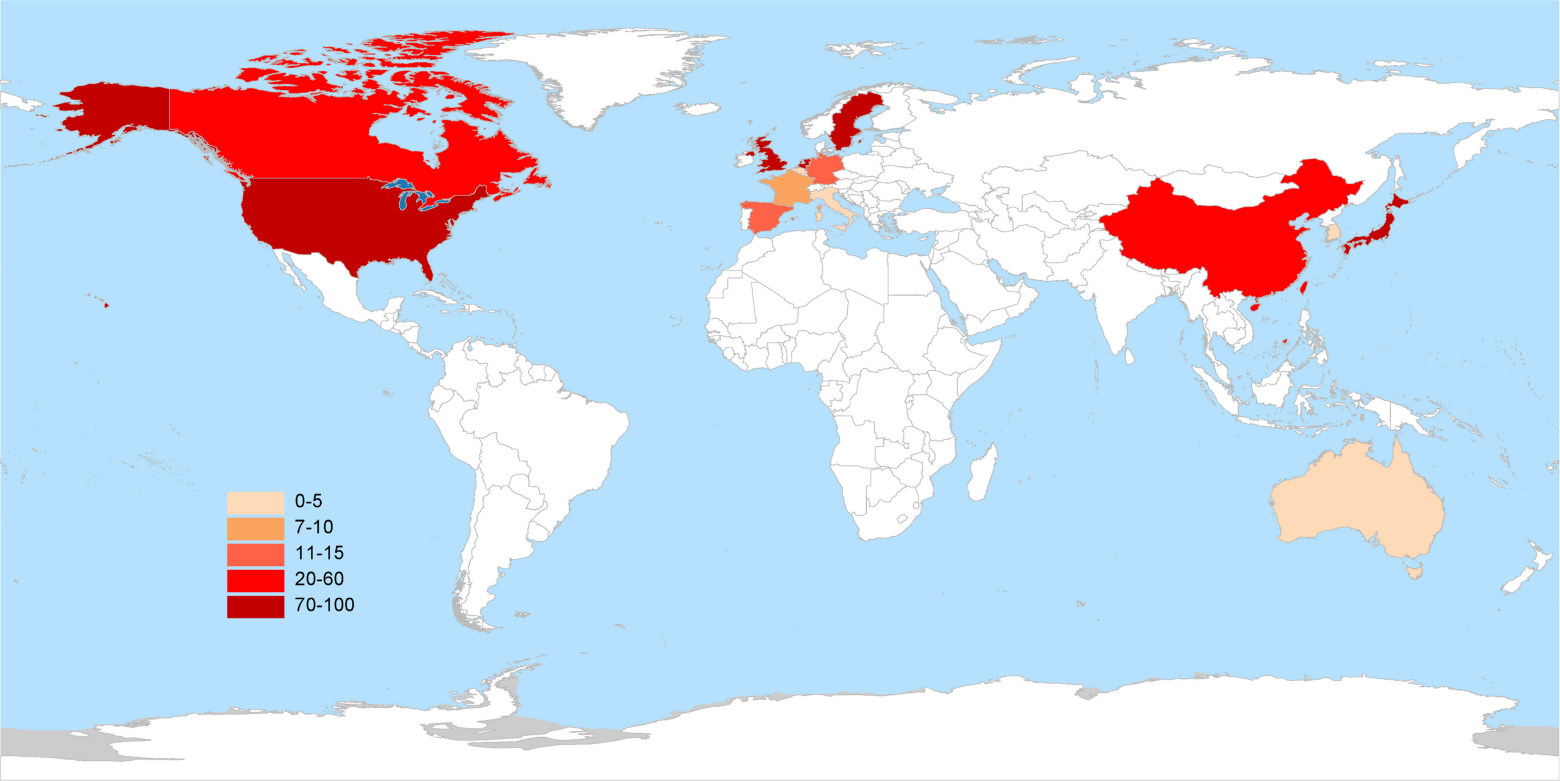
Global distribution of clinical trials for TCR-T cell therapy in cancer treatment.

**Figure 3:**
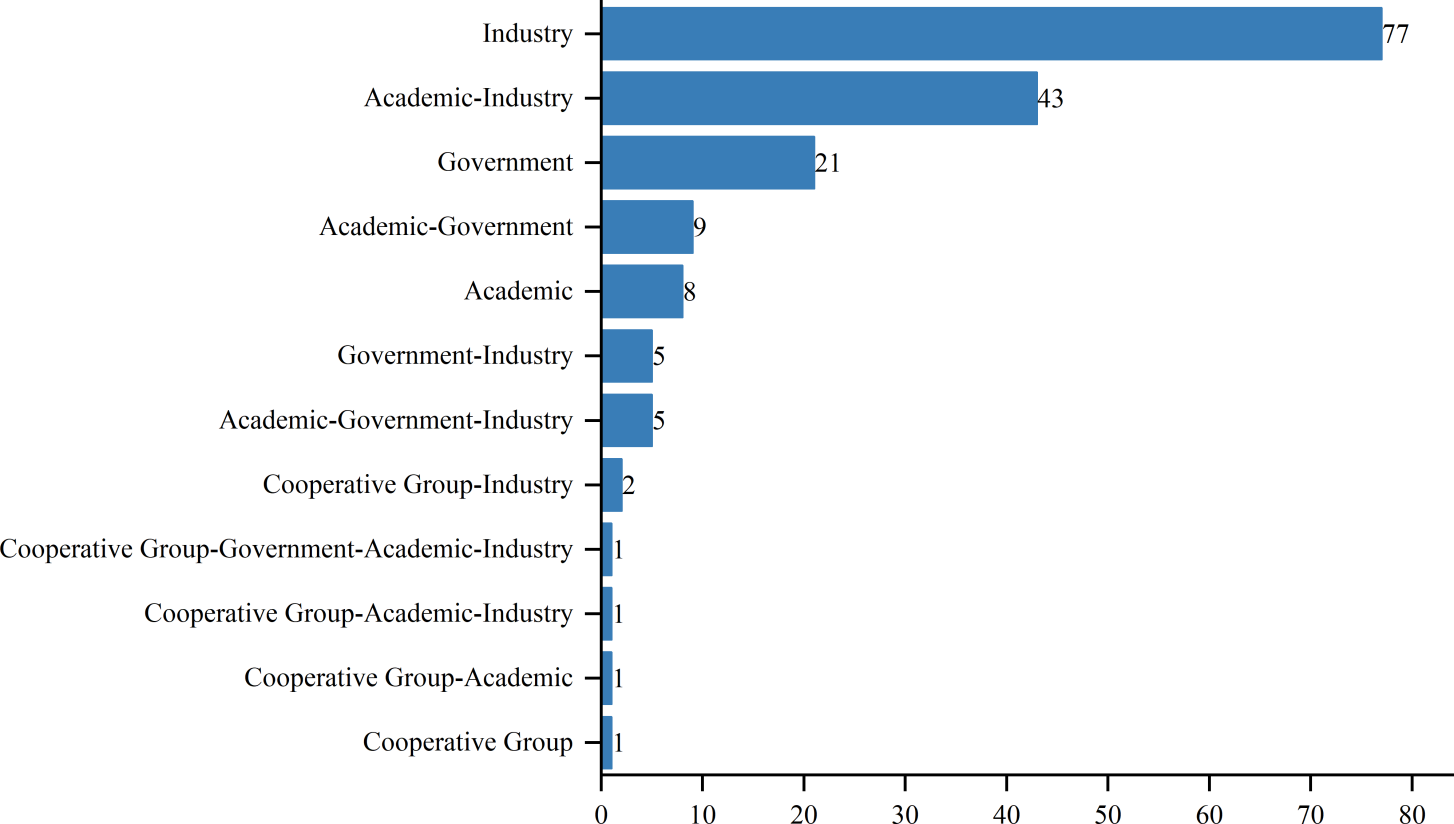
Funding types in research projects.

As of August 1, 2024, 417 interventional clinical trials were registered worldwide. Exclusions included 48 trials lacking a specified start date, 2 trials under “other” phases, 174 trials with unknown drug targets, and 19 trials with unspecified countries, leaving 174 trials for analysis. Trial registration began in 2006, with numbers increasing annually, peaking in 2019 and 2022 with 24 trials each year (**Figure 1**). Geographically, most trials were conducted on a single continent: North America hosted 84 trials (48.3%), Asia hosted 62 trials (35.6%), and Europe hosted 8 trials (4.6%). Multi-continental trials included one trial in Asia and North America (0.6%), four trials in Oceania, Europe, and North America (2.3%), and 15 trials in Europe and North America (8.6%) (**Figure 2**). Funding sources varied: 77 trials (44.3%) were industry-funded, 21 (12.1%) were government-funded, 8 (4.6%) were academically funded, and 1 (0.6%) was funded by a cooperative group. Additionally, 43 trials (24.7%) received combined academic and industry funding, nine (5.2%) from academic and government sources, five (2.9%) from government and industry sources, five (2.9%) from academic, government, and industry sources, two (1.1%) from a cooperative group and industry, one (0.6%) from a cooperative group and academia, one (0.6%) from a cooperative group, academia, and industry, and one (0.6%) from a cooperative group, government, academia, and industry (**Figure 3**).

### 3.2 Clinical trial phases and designs

**Figure 4:**
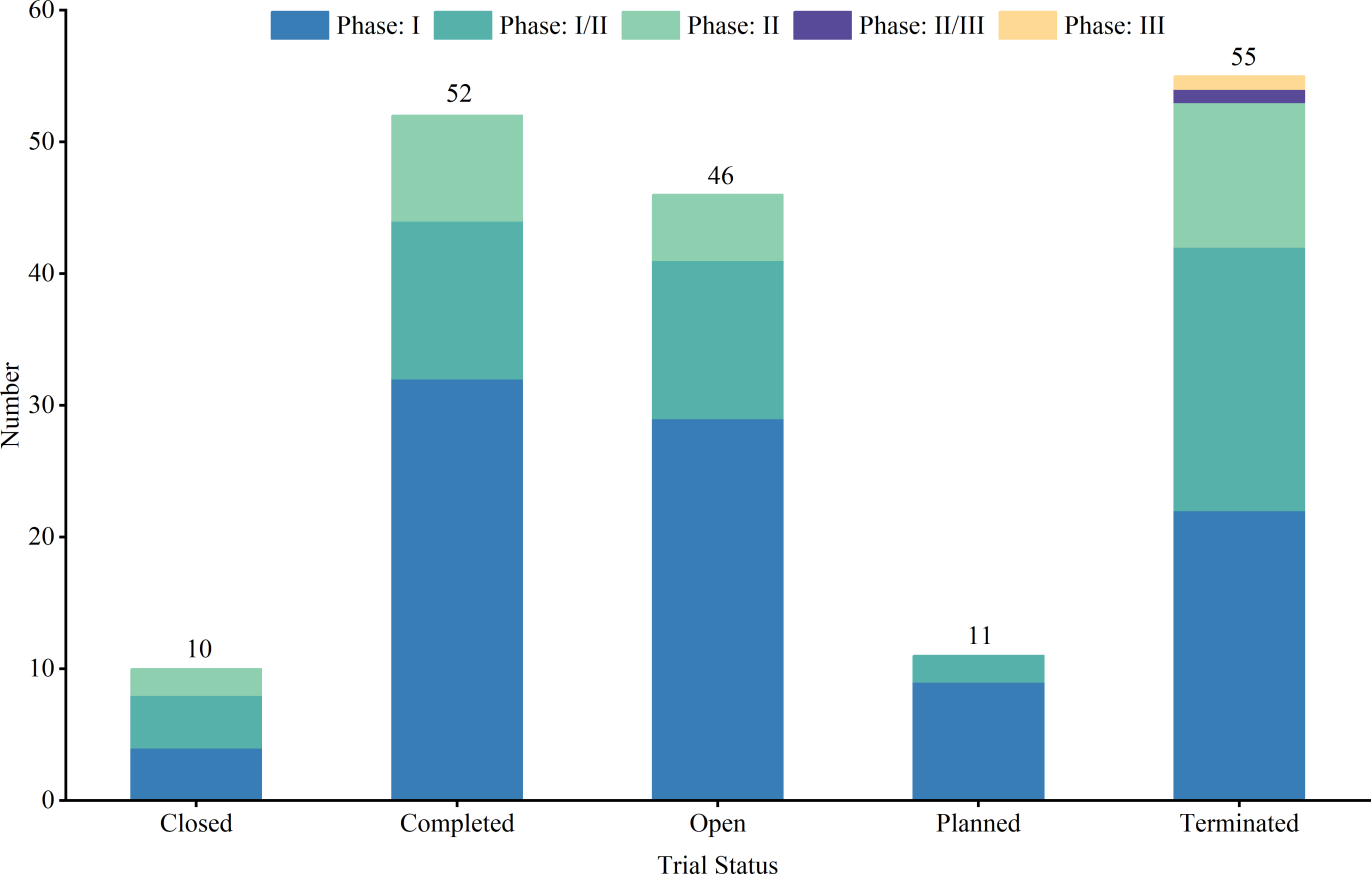
Trial phase and trial status distribution of clinical trials for TCR-T cell therapy in cancer treatment.

**Figure 5:**
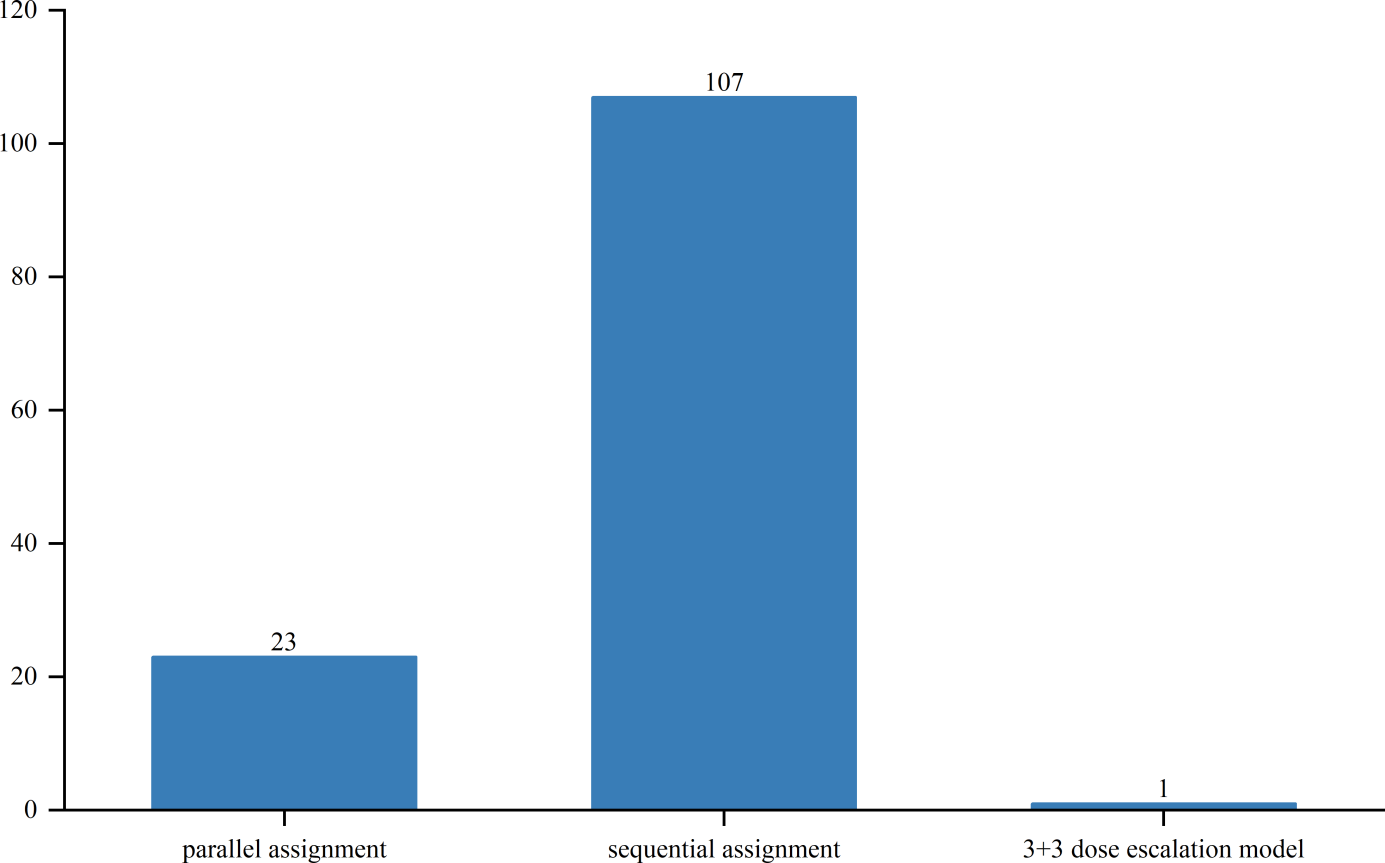
Allocation distribution of clinical trials for TCR-T cell therapy in cancer treatment.

**Figure 6:**
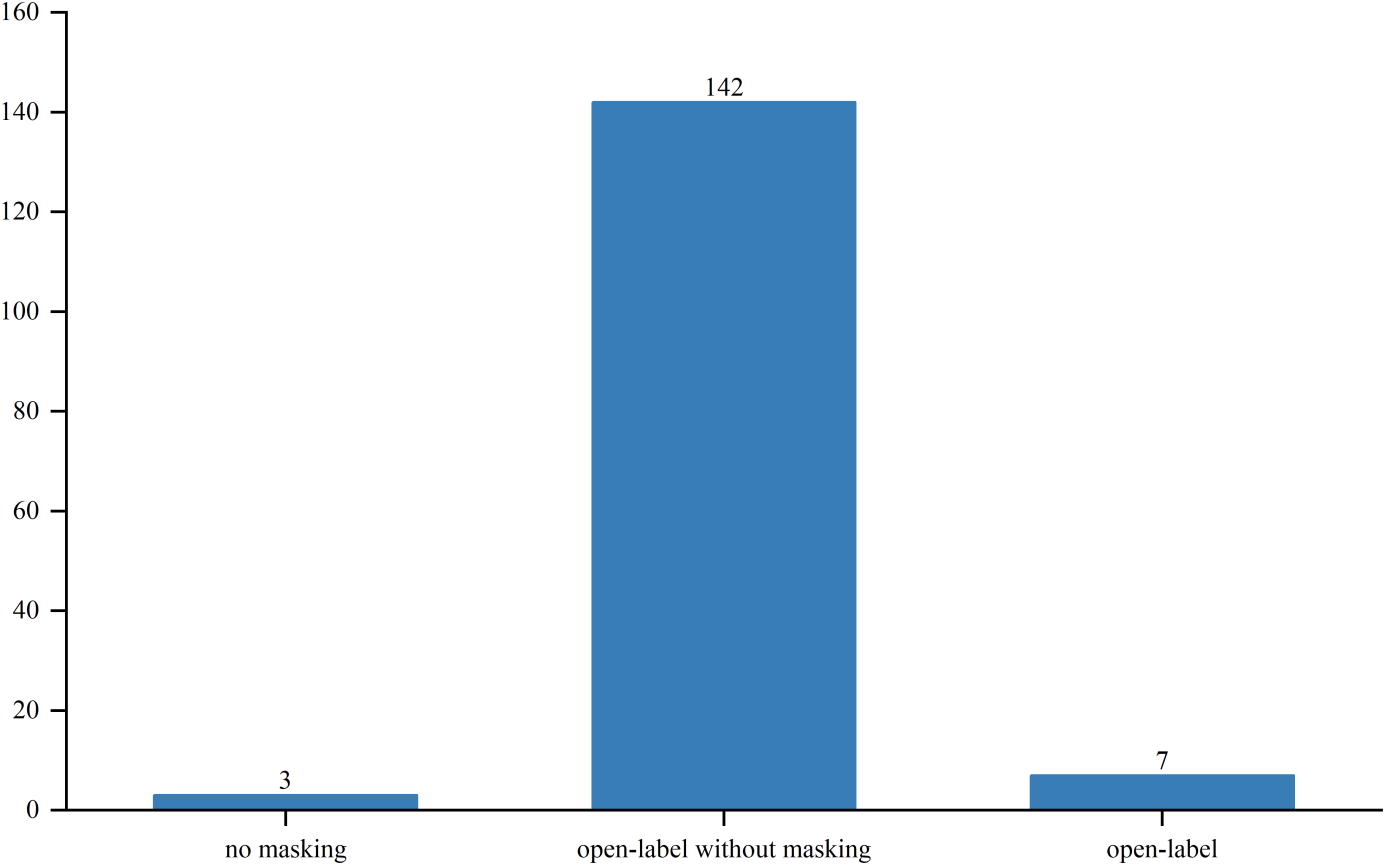
Masking distribution of clinical trials for TCR-T cell therapy in cancer treatment.

Out of the studied trials, 96 were in Phase I, 76 in Phase II and Phase I/II, and only 2 in Phase III and Phase II/III. The sole Phase III trial, a pivotal, open-label pilot study, focused on safety and efficacy. Trial statuses varied: 11 trials (6.3%) were closed, 11 (6.3%) planned, 43 (24.7%) ongoing, 51 (29.3%) terminated, and 52 (29.9%) completed (**Figure 4**). Allocation included three randomized and 30 non-randomized trials, with others unspecified. Of the three randomized trials, two were in Phase II and one in Phase I/II. Intervention models included 23 parallel assignments, 20 with sequential assignments, 107 with single-group assignments, 1 with a 3 + 3 dose escalation model, and 1 with crossover assignments; other models were unspecified (**Figure 5**). Regarding masking, 3 trials employed no masking, 142 were open-label without masking, and 7 were open-label; the masking details of the remaining trials were not specified (**Figure 6**).

### 3.3 Indications and target analysis in clinical trials

**Figure 7:**
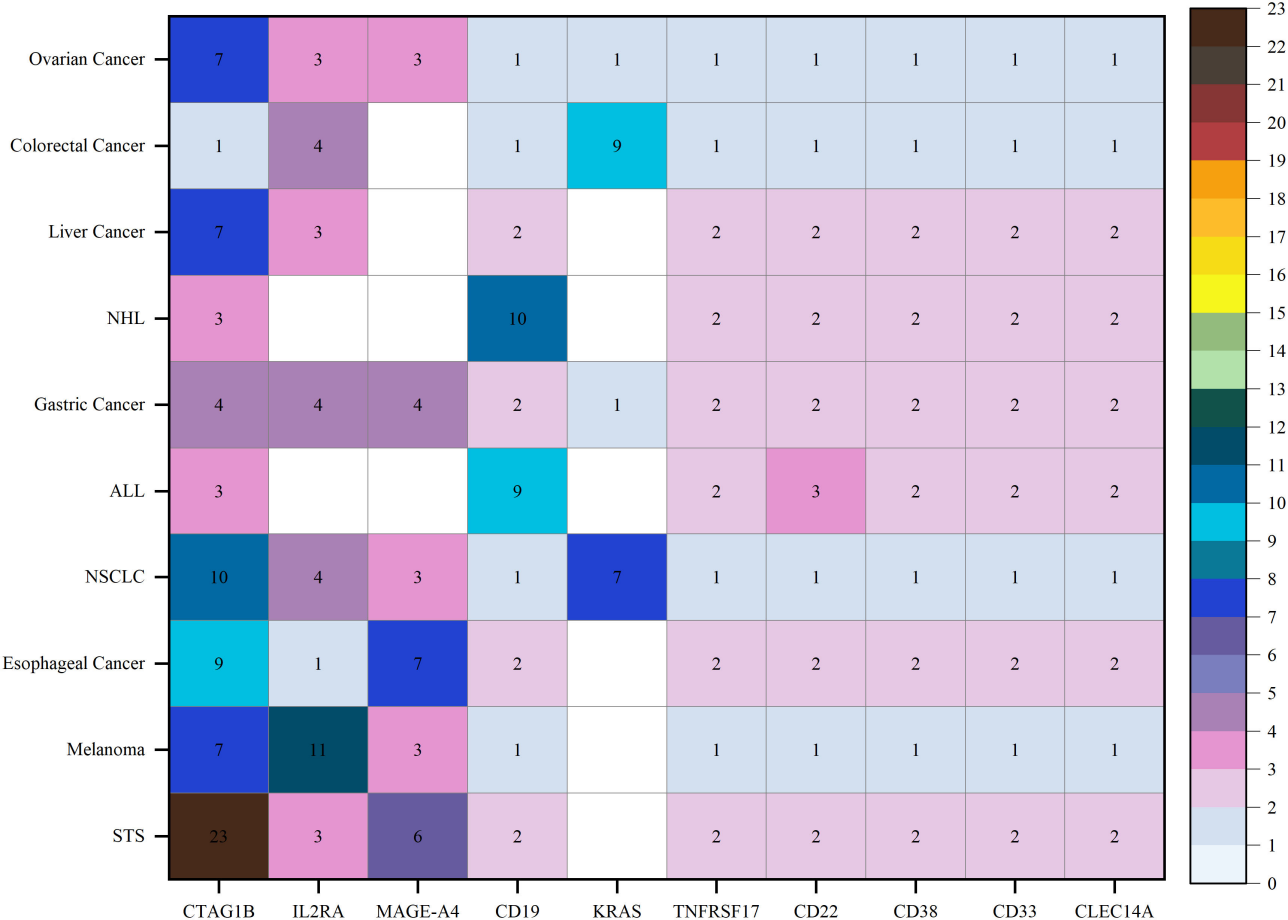
Disease and targets distribution of clinical trials for TCR-T cell therapy in cancer treatment.

In the evolving landscape of TCR-T cell therapy research, an analysis of 174 registered trials identified the 10 most frequently studied cancer types: soft tissue sarcoma (STS), melanoma, esophageal cancer, non-small cell lung cancer (NSCLC), ALL, gastric cancer, non-Hodgkin lymphoma (NHL), liver cancer, colorectal cancer, and ovarian cancer. Target analysis from these trials revealed that cancer-testis antigens were frequently studied, with cancer-testis antigen 1B (CTAG1B, also known as NY-ESO-1) emerging as a prominent target in STS and showing notable relevance in NSCLC, esophageal cancer, melanoma, liver cancer, and ovarian cancer. The MAGE family members, particularly MAGE-A4, exhibit high targeting potential in STS and esophageal cancer. Interleukin-2 receptor alpha chain (IL2RA) was predominantly targeted in melanoma, with moderate targeting in other cancers. CD19 was primarily targeted in ALL and NHL, while KRAS was frequently targeted in colorectal cancer. Other targets, including TNF receptor superfamily member 17 (TNFRSF17), CD22, CD38, CD33, and C-type lectin domain containing 14A (CLEC14A), demonstrated consistent but lower targeting potential across cancers (**Figure 7**, **Supplementary Table S1**).

### 3.4 Mechanism of action

The mechanisms of action of the main drugs tested in our clinical trials varied, predominantly focusing on T-cell stimulation and immuno-oncology therapies. A considerable majority, 80.5% (140 trials), involved solely T-cell stimulators and immuno-oncology therapy. Additionally, combinations of T cell stimulants with IL-2 agonists and unidentified pharmacological activities comprised 10.3% (18 trials), while combinations with immune checkpoint inhibitors constituted 3.4% (six trials), and another 3.4% (six trials) combinations with PD-1 antagonists. Furthermore, 1.1% (two trials) combined T-cell stimulators, immuno-oncology treatments, and immunostimulants. One trial (0.6%) included a combination of T-cell stimulators, immuno-oncology therapy, and a B-cell maturation antigen antagonist. Finally, one trial (0.6%) explored the combination of T-cell stimulators, immuno-oncology treatments, and gene editing technologies.

## 4 Discussion

In recent years, the field of TCR-T cell therapy for cancer treatment has gained widespread attention and achieved significant progress, highlighted by the annual increase in clinical trials from 2006 to 2024, with a peak occurring between 2019 and 2024. This trend not only underscores the growing interest but also reflects the increasing confidence in the clinical potential of this therapy, suggesting a maturation of the technology and a shift toward its application in more challenging cancer types. As the body of clinical and preclinical research continues to grow, it becomes essential to understand the factors that influence the efficacy of this therapy, such as patient genetics, tumor heterogeneity, and immune microenvironment characteristics. These factors can shape therapy outcomes and must be taken into account in trial designs and therapeutic approaches.

Ethnic and racial differences have been shown to influence the efficacy of immunotherapies, including TCR-T cell therapy, due to underlying genetic variations in HLA types and tumor antigen presentation (20). This underscores the need for diverse patient enrollment in clinical trials to mitigate disparities in treatment outcomes and to ensure that therapies are broadly applicable across different populations. The inclusion of diverse populations can provide a more comprehensive understanding of potential genetic influences on treatment response, which is crucial for the development of personalized cancer therapies. The diversity in funding sources reflects the convergence of multiple sectors in advancing TCR-T therapies. Industry-funded trials, which often constitute the majority, tend to be more resource-intensive, with a focus on commercialization, which contributes to robust patient recruitment and comprehensive data collection (21). Conversely, government and academically funded trials often emphasize innovation and novel therapeutic approaches that may not yet be commercially viable, and these trials may encounter limitations in patient recruitment and resources (22). A synergistic approach combining these different funding sources has the potential to strike a balance between practicality and innovation, optimizing both clinical trial efficiency and therapeutic development.

Trial status analysis further highlights the dynamic and ongoing nature of research in TCR-T cell therapy. While the completion of trials provides immediate insights, ongoing and planned trials indicate the field’s continued momentum, especially in overcoming early-stage obstacles such as patient recruitment and interim efficacy (23). Notably, TCR-T cell therapy has demonstrated efficacy in multiple Phase I and II trials, particularly against refractory and recurrent cancers, underscoring its potential for transforming treatment in difficult-to-treat cancers. Technological innovations, such as CRISPR-based gene editing and advanced T cell manufacturing techniques, play pivotal roles in enhancing both the safety and precision of TCR-T therapies. These advancements not only improve tumor targeting but also reduce off-target effects, laying the groundwork for more successful outcomes in future clinical trials. In addition to these technological innovations, insights gained from terminated trials are also invaluable, helping to refine future research. Each failed trial provides lessons on patient selection, target optimization, and therapeutic protocols, helping to reshape clinical strategies and ensure that future trials are more effectively designed. This continuous feedback loop between research, trial outcomes, and technological advancement is crucial for the ongoing evolution of TCR-T cell therapies, offering renewed hope for patients who are presented with limited treatment options.

In clinical trials of TCR-T cell therapies, several targets have garnered research interest due to their tumor cell-specific expression and potential to elicit a robust immune response. Among these targets, MAGE-A4 stands out as a significant cancer-testis antigen, well-known for its high immunogenicity and widespread expression in various solid tumors. The selective expression of MAGE-A4 in tumor cells, with minimal expression in normal tissues, makes it an optimal target for TCR-T cells that are genetically engineered for high-affinity tumor recognition. This selective targeting enhances treatment efficacy by reducing off-target effects and improving tumor eradication (24). In both *in vitro* and *in vivo* models, the modified TCR-T cells effectively recognized and killed tumor cells expressing MAGE-A4. In the mouse model, these cells showed strong anti-tumor activity and significantly inhibited tumor growth. This lays a strong foundation for the clinical application of MAGE-A4-targeted TCR-T cell therapy (25). A Phase I clinical trial (NCT03247309) validated this approach, where MAGE-A4-specific TCR-T cells significantly reduced tumor size in patients with refractory solid tumors. The success of this trial highlights the clinical potential of MAGE-A4-specific TCR-T cells in combating hard-to-treat cancers. In another trial, NCT03132922, an overall response rate (ORR) of 24% was reported, with a response rate of 44% in patients with STS. These findings, when contrasted with the 9% ORR in other cancers, underscore the tumor-specific efficacy of the antigen and the necessity of ongoing research to optimize its use. Preclinical studies also reinforce these outcomes, further substantiating the rationale for MAGE-A4-specific TCR-T cell therapies in challenging cancer types and underscoring the potential of MAGE-A4-specific TCR-T cells in treating challenging cancers (26).

Similarly, other tumor antigens like CTAG1B have also shown great potential in advancing immunotherapy. The researchers found that the modified TCR-T cells specifically recognized CTAG1B, activated T cells, and significantly inhibited cancer cell proliferation in an *in vitro* model (27). This suggests that CTAG1B could be a key target for solid tumor treatment. CTAG1B has emerged as a primary target for TCR-T therapy, with clinical trials demonstrating its strong anti-tumor activity, especially in STS (28). The favorable response rates observed in these trials ranged from 35.7% to 66.7%, highlighting the importance of continued exploration of CTAG1B-targeted therapies, especially in cancers with limited therapeutic options (29). Additionally, in a clinical trial (NCT04318964) investigating TCR-T cell therapy targeting CTAG1B, an ORR of 41.7% was observed across breast, liver, ovarian, and soft tissue sarcoma cancers. These consistent findings suggest that targeting well-characterized antigens like CTAG1B can significantly impact the therapeutic landscape for cancer, reinforcing the importance of ongoing research and development in TCR-T cell therapies to maximize clinical outcomes.

Beyond cancer-testis antigens, immune cell surface markers such as CD19 have been extensively explored for their therapeutic potential in immunotherapy, particularly in B-cell malignancies. CD19 is predominantly expressed on B cells and plays a crucial role in diseases such as ALL and diffuse large B-cell lymphoma (30). CD19-specific TCR-T cells have shown strong anti-tumor activity in *in vitro* models. These cells effectively recognized and killed CD19-expressing tumor cells, activating T cell-mediated immune responses (31). In a clinical trial (NCT03156101) evaluating TCR-T cell therapy targeting the CD19 molecule, the ORR was 77% in patients with ALL and non-Hodgkin’s lymphoma. Complete remission was achieved in 6 out of 14 patients at the 3-month follow-up, indicating a strong therapeutic potential of CD19-targeted therapies in hematologic malignancies. The role of signaling molecules and immune modulators, such as IL2RA, has also gained prominence in enhancing TCR-T therapies. As part of the IL-2 receptor complex, IL2RA acts as an early activation marker that promotes T-cell proliferation and survival through IL-2 binding. Strategic modulation of IL2RA expression in TCR-T therapies can significantly enhance treatment efficacy by increasing T-cell sensitivity to IL-2 within the tumor microenvironment. IL2RA-specific TCR-T cells effectively induced tumor cell apoptosis in *in vitro* experiments and demonstrated strong tumor suppression in *in vivo* studies (32). This highlights IL2RA’s potential in immuno-oncology therapy. Studies have also shown that overexpressing IL2RA in specific tumor microenvironments can markedly improve anti-tumor activity by promoting T-cell proliferation and persistence (33). This strategy is supported by *in vitro* evidence showing improved T-cell function following IL2RA upregulation, suggesting that future clinical trials should explore this approach to enhance TCR-T therapies in hostile tumor microenvironments (34).

In addition to cancer-testis antigens, mutations in genes such as KRAS have emerged as promising targets for TCR-T therapies, particularly in cancers such as pancreatic and colorectal cancers (NCT03190941). KRAS mutations represent a critical area of focus because they are frequently associated with tumor proliferation and survival (35). Specifically, the KRAS-G12V mutation generates a 9-peptide neoantigen that can be effectively recognized by specific TCRs, triggering the activation of TCR-T cells to secrete cytokines and kill tumor cells. Preclinical studies, including mouse models, have demonstrated that KRAS-G12V-specific TCR-T cells exhibit significant anti-tumor activity. This effect is further enhanced when combined with PD-1 antibodies, suggesting that KRAS-targeted TCR-T cell therapies could lead to long-term tumor remission (36). Additionally, TCR-T cell therapies targeting the KRAS G12D mutation have shown strong performance in both *in vitro* and *in vivo* models. KRAS G12D-specific TCR-T cells effectively recognize and kill cancer cells carrying this mutation. In mouse models, these cells showed strong tumor growth inhibition. AFNT-212, a TCR-T cell product targeting KRAS G12D, has shown good safety and efficacy (37). Although research is still in its early stages, the success of these studies suggests that KRAS-targeted TCR-T cell therapies could lead to long-term tumor remission in patients with limited treatment options (38). As the field evolves, ongoing research into KRAS mutations and their role in cancer progression will continue to inform the development of TCR-T cell therapies, potentially expanding their application to a broader range of cancer types.

Beyond targeting mutations, addressing the migratory and invasive properties of tumor cells has emerged as another critical strategy in cancer immunotherapy. The gene ARHGAP45 has gained increasing attention due to its association with cytoskeleton regulation and cell migration. ARHGAP45 plays a pivotal role in regulating tumor cell migration and invasion, processes integral to cancer metastasis. By influencing the cytoskeleton dynamics via Rho family GTPases, ARHGAP45 controls cell shape, adhesion, and motility, making it an ideal therapeutic target for inhibiting metastasis. In the context of TCR-T cell therapy, targeting ARHGAP45 may help to inhibit tumor invasiveness and metastatic potential. For example, by specifically identifying and targeting ARHGAP45-expressing tumor cells, TCR-T cells can directly attack these highly migratory and invasive cancer cells, thereby reducing the risk of metastasis and improving overall therapeutic outcomes. The potential of this strategy has been preliminarily demonstrated in several early clinical studies (19). Additionally, targeting ARHGAP45 may work synergistically with other anti-tumor therapies. For instance, ARHGAP45 inhibition could enhance the effects of other immunotherapies such as immune checkpoint inhibitors, by improving the immune system’s ability to recognize and destroy tumors by reducing the invasiveness of tumor cells. Thus, combining TCR-T cell therapy with ARHGAP45-targeted approaches may offer a more comprehensive and effective therapeutic strategy (39).

To maximize the efficacy of TCR-T therapies, ongoing clinical trials have increasingly integrated advanced immunotherapy approaches and combination treatments. The primary focus of many trials is the use of T cell stimulators to enhance the proliferation and survival of genetically engineered TCR-T cells. T-cell stimulators work by interacting with co-stimulatory molecules on T cell surfaces, boosting T-cell function and extending their *in vivo* survival, thereby increasing their tumor-killing capabilities (40). These stimulators also help T cells overcome inhibitory signals within the tumor microenvironment, enabling them to penetrate tumors more effectively and sustain therapeutic impact (41). Clinical trials have demonstrated that T-cell stimulators can be used both independently and in combination with therapies such as immune checkpoint inhibitors, with OX40 stimulators showing synergistic effects when combined with checkpoint inhibitors, leading to improved overall survival in patients (42). This multi-faceted approach suggests that T-cell stimulators hold significant promise, particularly in combination with other immunotherapies, to enhance treatment outcomes across a broad range of cancer types.

In parallel, immuno-oncology has emerged as a crucial strategy in TCR-T cell therapy. This approach has shown significant efficacy across various tumor types, particularly when combined with T-cell stimulators, to further optimize the effectiveness of TCR-T cell therapy. Immuno-oncology therapies enhance the anti-tumor cactivity of the immune system through various immunomodulatory strategies, primarily including immune checkpoint inhibitors and cytokine therapy. Immune checkpoint inhibitors operate by restoring the anti-tumor activity of T cells, blocking inhibitory signals on the surface of T cells—such as those in the PD-1/PD-L1 pathway—thereby boosting the efficacy of TCR-T cell therapy. This approach has proven particularly effective in counteracting immunosuppressive factors within the tumor microenvironment, allowing TCR-T therapies to produce better clinical outcomes (43). Additionally, immuno-oncology therapies can reduce the negative impact of these suppressor cells on the anti-tumor immune response by modulating regulatory T cells (Tregs) and tumor-associated macrophages. By doing so, these therapies synergize with TCR-T cell therapies to further enhance the anti-tumor capacity of T cells, leading to improved overall efficacy. This synergistic effect between TCR-T therapy and immuno-oncology strategies has been demonstrated in multiple clinical trials, further broadening the therapeutic applicability of TCR-T cell therapies (39). By combining multi-targeted immunotherapies, it is possible to enhance overall efficacy, leading to improved treatment responses in various cancer types.

This study provides a comprehensive analysis of global clinical trial data on TCR-T cell therapy, covering various cancer types and trial phases. The extensive data coverage and detailed tumor antigen analysis offer valuable insights for optimizing treatment design and target selection, while also identifying key trends in research and clinical practice, such as the potential for multi-targeting and combination therapies. However, this study has some limitations. First, a significant portion of the data is derived from Phase I and II clinical trials, which generally have small sample sizes and lack long-term follow-up, limiting the ability to thoroughly assess the long-term efficacy and safety of TCR-T cell therapy. To address these issues, future research should prioritize the inclusion of more diverse patient cohorts and offer extended follow-up periods. This can help validate the preliminary findings from earlier phases, enabling more robust assessments of efficacy and safety across different cancer types and patient demographics. Additionally, the establishment of international collaborative networks could facilitate the pooling of data from multiple trials, providing more comprehensive insights into the long-term effects of TCR-T cell therapy. Another limitation lies in the variability in trial designs across different studies, which complicates data integration. Differences in dosing regimens, patient selection criteria, and outcome measures can result in heterogeneity, which makes direct comparisons challenging. To mitigate this, future studies should aim to standardize trial protocols where possible or, at the very least, ensure consistent reporting of key trial variables. This would improve data comparability and allow for more accurate meta-analyses that could help refine TCR-T cell therapy protocols. Furthermore, the analyses are primarily based on clinical trial data from the Trialtrove database, which, despite its comprehensiveness, may still miss information from underreported or unregistered trials. Inconsistent clinical trial registration practices across different regions may also lead to the exclusion of certain trial data, potentially affecting the comprehensiveness of the conclusions. Future studies should aim to supplement database searches with additional sources of information, such as unpublished trial results, or by incorporating data from real-world evidence (RWE) studies and expanded access programs. RWE can provide insights into how TCR-T cell therapies perform in non-trial settings, further enhancing our understanding of their effectiveness and safety.

Given these considerations, it is clear that while TCR-T cell therapy holds immense promise, its transition from experimental stages to clinical reality requires the addressing of several challenges. Larger trials, longer follow-up periods, and more standardized data will contribute to more definitive conclusions regarding the potential of TCR-T cell therapies. Future research must also focus on overcoming the hurdles associated with tumor heterogeneity and immune escape mechanisms, which limit the broad applicability of TCR-T therapy. Moreover, optimizing the scalability of manufacturing processes and improving the management of adverse events is critical for the widespread adoption of TCR-T therapies in clinical oncology. By addressing these challenges, the full potential of TCR-T cell therapy as a powerful weapon against refractory cancers can be unlocked, advancing the field of cancer immunotherapy.

In conclusion, TCR-T cell therapy has emerged as a highly promising approach to cancer immunotherapy, particularly for refractory and relapsed malignancies. By engineering T cells to express tumor-specific TCRs, this therapy enhances the ability of the immune system to identify and eliminate cancer cells. Despite its significant therapeutic potential, several challenges remain, including issues related to tumor heterogeneity, immune escape, and the complexities of manufacturing. To fully realize the potential of TCR-T cell therapy, future research should focus on optimizing TCR design, exploring multi-targeted and combination therapies, reducing the risk of off-target effects, and improving the scalability of production processes. These advancements will be critical for expanding the application of TCR-T therapies across a broader range of cancer types, helping to solidify their role in modern cancer treatment.

## 5 Conclusions

TCR-T cell therapy has demonstrated great potential in cancer treatment, especially for advanced tumors, and has made significant progress. However, the process of clinical translation still faces multiple challenges, including the complexity of antigen selection and immune escape mechanisms. In addition, the complexity of the manufacturing process and the high cost of treatment also limit the large-scale application of this therapy. Future research should focus on optimizing TCR design, reducing the risk of off-target effects, developing multi-target and combination therapy strategies, and improving large-scale production technologies. These advances will help move TCR-T cell therapy from the laboratory to wider clinical applications, ultimately providing a more effective and safer treatment option for cancer patients.
